# Diagnostic Accuracy of Interleukin-17A for Internal Derangements of Temporomandibular Joints in Patients with Spondyloarthritis

**DOI:** 10.3390/biomedicines14020424

**Published:** 2026-02-13

**Authors:** Ana-Marija Laškarin, Vedrana Drvar, Stjepan Špalj, Gordana Laskarin, Emina Babarović, Tatjana Kehler, Viktor Peršić, Nikša Dulčić

**Affiliations:** 1 School of Dental Medicine, University of Zagreb, Gundulićeva 5, 10000 Zagreb, Croatia; 2 Clinical Department of Laboratory Diagnostics, Clinical Hospital Center Rijeka, Vjekoslava Dukića 7, 51000 Rijeka, Croatia; vedranadrvar@gmail.com; 3 Department of Orthodontics, Faculty of Dental Medicine, University of Rijeka, Krešimirova 40/42, 51000 Rijeka, Croatia; stjepan.spalj@fdmri.uniri.hr; 4 Department of Dental Medicine, Faculty of Dental Medicine and Health, J. J. Strossmayer University of Osijek, Crkvena 21, 31000 Osijek, Croatia; 5 Department of Physiology, Immunology and Pathophysiology, Faculty of Medicine, University of Rijeka, B. Branchetta 20, 51000 Rijeka, Croatia; gordana.laskarin@medri.hr; 6 Hospital for Medical Rehabilitation of Hearth and Lung Diseases and Rheumatis “Thalassotherapia-Opatija”, M. Tita 180, 51410 Opatija, Croatia; tatjana.kehler@ri.t-com.hr (T.K.); viktor.persic@medri.uniri.hr (V.P.); 7 Clinical Department of Pathology and Cytology, Clinical Hospital Center, University of Rijeka, Krešimirova 42, 51000 Rijeka, Croatia; emina.babarovic@medri.uniri.hr; 8 Department of Medical Rehabilitation, Faculty of Medicine, University of Rijeka, B. Branchetta 20, 51000 Rijeka, Croatia; 9 Department of Removable Prosthodontics, School of Dental Medicine, University of Zagreb, Gundulićeva 5, 10000 Zagreb, Croatia; dulcic@sfzg.hr

**Keywords:** interleukin-17A, interleukin-12/interleukin-23 protein 40, matrix-metalloproteinase 3, spondyloarthritis, temporomandibular disorders, temporomandibular internal derangements, temporomandibular joints

## Abstract

**Objective**: The oral cavity is the beginning of the digestive tract and the composition of saliva could indicate immune events in the gut and joints. The objective of this research was to evaluate the diagnostic accuracy of salivary interleukin (IL)-17A for temporomandibular joint (TMJ) internal derangements (IDs) in patients with spondyloarthritis (SpA). **Methods**: SpA disease activity was assessed using the Bath Ankylosing Disease Activity Index (BASDAI), Ankylosing Spondylitis Disease Activity Score (ASDAS) and Disease Activity Index for Psoriatic Arthritis (DAPSA). Salivary cytokines were analyzed using enzyme-linked immunosorbent assay. TMJ conditions were evaluated using The Diagnostic Criteria for Temporomandibular Disorder (DC/TMD) protocol. A symptomatic TMJ-ID group with intracapsular arthralgia (*n* = 64) and asymptomatic TMJ-ID group without intracapsular arthralgia (*n* = 50), regardless of joint sounds, were compared with controls (healthy TMJs, *n* = 86). **Results**: Women were more prevalent and salivary IL-17A concentration was higher in both ID groups than in controls. Salivary IL-17A levels positively correlated with erythrocyte sedimentation rate, anti-streptolysin-O titer, salivary IL-12/23 p40 and matrix metalloproteinase-3 levels, sore and swollen joint counts, BASDAI, chronic TMJ pain and anxiety. IL-17A demonstrated diagnostic accuracy for currently symptomatic (cutoff, 11 pg/mL) and asymptomatic (cutoff, 11.6 pg/mL) TMJ-ID vs. controls. Patients with IL-17A levels above these cutoffs more frequently exhibited disc displacement with reduction and degenerative TMJ disease, higher self-reported spinal pain and higher SpA activity, as assessed by ASDAS, than patients with IL-17A levels ≤ cutoffs. TMJ-related headache and somatization contributed to greater TMJ pain in those with IL-17A > cutoffs, when compared with dichotomous controls. **Conclusions**: Salivary IL-17A concentration provides an accurate laboratory marker of SpA activity and enables the diagnosis of both currently symptomatic and asymptomatic TMJ-IDs in patients with SpA.

## 1. Introduction

Spondyloarthritis (SpA) is a diverse group of chronic inflammatory diseases that develop as a result of environmental factors in individuals with a genetic predisposition, with an age of onset of 45 years [1]. Although SpA affects the spine (axial SpA) and peripheral joint structures (peripheral SpA) in varying proportions, all subgroups of SpA (non-radiologic axial SpA, radiologic axial SpA, psoriatic arthritis, reactive arthritis, and SpA linked to inflammatory bowel diseases and undifferentiated forms of SpA) exhibit similar clinical features in adults [2,3]. Intestinal dysbiosis serves as a central driver of inflammation in SpA and psoriasis [4]. The oral cavity is the beginning of the digestive tract and harbors a physiologically large number of microbial species [5], which colonize other parts of the digestive tract, highlighting the direct connection between the mouth and intestines [4]. Saliva is considered an excellent medium for assessing the intestinal environment owing to its abundance of enzymes and cytokines, particularly in cases of intestinal dysbiosis [3,4]. However, little is known about interleukin (IL)-12/23 protein (p)40, IL-17A, matrix metalloproteinase-3 (MMP-3) and tumor necrosis factor alpha (TNF-α) concentrations in the saliva of patients with SpA. Protein p40 is a free common subunit of IL-12 and IL-23, which is produced by dendritic cells (DCs) in dysbiosis and represents an early sign of host–pathogen interaction, which precedes the secretion of whole IL-12 (p75) [6], favouring IL-23 production in SpA [7]. IL-23 is indispensable for the differentiation of Th17+ lymphocytes and innate lymphoid cell 3 (ILC3), which secrete IL-17A in the inflamed intestinal mucosa [7,8]. Dendritic cells [9] and ILC3 [10] leave the mucosa and settle in lymph nodes, entheses, and joints, where they continue IL-23 or IL-17A cytokine production and T-cell recruitment in patients with SpA [7,11], supporting the concept of an oral–intestinal–joint axis in the pathogenesis of SpA [10]. In entheses and joints, the secretion of the potent inflammatory TNF-α [7] and MMPs were stimulated from connective tissue and immune cells, whose activation is responsible for extracellular matrix degradation and pain [12,13].

Intracapsular pain in the temporomandibular joint (TMJ) can be attributed to disc dislocation only after resolution of synovitis or capsular impingement [14], although the inflammation is a common cause of temporomandibular disorders (TMDs) [15] including internal derangements (IDs) [16], particularly in patients with autoimmune diseases [3,17]. IL-17A and its receptors were found in TMJ synovial fibroblasts isolated from patients with TMD [17], but studies on the role of salivary IL-17A in the diagnosis of TMD in SpA patients are lacking.

TMDs cause inflammatory changes in intra-articular and extra-articular connective tissue and are divided into two main groups using the Diagnostic Criteria for TMD (DC/TMD) protocol [16,18]. The first group comprises TMJ-IDs, which are associated with changes in the position of the intra-articular disc relative to the mandibular condyle and temporal fossa, and degenerative processes, respectively. TMJ-IDs exhibit clicking, crepitus, and/or limited jaw movement [19]. The second group comprises extra-articular disorders related to muscle and joint pain and headache, and includes local myalgia, myofascial pain, myofascial pain with spreading, headache attributed to TMDs, and arthralgia [19]. Both groups can be painless or can cause pain but in different joint structures [19].

The DC/TMD identifies clinically relevant TMJ-ID based on functional clinical signs and symptoms (joint noise with or without intermittent locking or limited mouth opening without joint noise) [19]. Today, magnetic resonance imaging (MRI) is considered the “gold standard” for the diagnosis of TMJ-IDs such as structural disc disorders that may or may not be associated with symptoms, and painful joint effusion, bone marrow edema, and even degenerative TMJ disease [16,20].

The hypothesis of this study was that salivary IL-17A concentration affects TMJ-IDs in patients with SpA. This study aimed to measure salivary IL-17A concentrations in patients with SpA and assess the diagnostic significance of IL-17A for symptomatic (with intracapsular arthralgia) and asymptomatic (without intracapsular arthralgia) IDs, as well as clinical and laboratory assessment of SpA activity.

## 2. Materials and Methods

### 2.1. Patients

In this cross-sectional study with consecutive sampling, Caucasian patients with SpA aged between 18 and 80 years (*n* = 200) were recruited. SpA was diagnosed based on clinical, laboratory and imaging findings according to the criteria of the Assessment of SpondyloArthritis International Society [1]. Patients were recruited from the rheumatology outpatient clinic of the Special Hospital for Medical Rehabilitation of Heart, Lung, and Rheumatism, “Thalassotherapia-Opatija”, Opatija, Croatia between 10 February 2021 and 28 February 2023.” (Figure S1).

Patients with SpA presented with cervical/axial pain (inclusion criteria) and peripheral joint pain of varying extent and passed rheumatological examination were included in this study. The exclusion criteria were patients with other rheumatic diseases, acute infection, history of malignant disease in the last 5 years, unregulated diabetes (fasting glucose above 11 mmol/L), arterial blood pressure > 160/100 mmHg, heart failure (New York Heart Association Criteria grade III and IV) [21], renal failure with filtration rate < 25 mL/min/1.73 m^2^), liver lesions (alanine transaminase, aspartate transaminase, and gamma-glutamyl transferase values exceeding the upper limit by threefold or more), recent trauma/bone fractures, or congenital/developmental disorders of TMJs. Patients had been taking medications from the non-steroidal anti-rheumatic drug and/or conventional disease-modifying anti-rheumatic drug groups as directed by a rheumatologist. No one used biological therapy, targeted synthetic disease-modifying anti-rheumatic drugs, or changed therapy regimen for the purpose of this study. After clinical examination, patients underwent laboratory evaluation. If a patient with SpA met the conditions for inclusion in this study, the rheumatologist advised the patient to have a TMJ examination for further assessment of SpA activity as part of the research protocol (Figure S1). Upon expressing willingness to participate, the patient was referred to the neighbouring general outpatient office and informed about the method of examining the TMJs (Axis I and II of the DC/TMD protocol) [19] and saliva sampling, which is painless, without harmful radiation or additional invasive procedures and could be performed immediately, lasting no longer than 10 min. After completion of these procedures, the patient’s active participation in this study ended. Participants were informed that the data obtained may have practical and scientific value and could be published in scientific journals. Patient identities remained anonymous. Participation in this study was voluntary, with no guarantee of direct benefit. Patients were informed that they could withdraw from this study at any time without providing a reason and without any health-related or legal consequences, and that their standard rheumatologic care would continue unchanged. By signing the Informed Consent Form, patients guaranteed their voluntary consent to examination of the TMJs, sampling and analysis of pro-inflammatory substances in saliva using enzyme-linked immunosorbent assay, and that members of the research team have access to medical records, and that they can conduct statistical processing and publish the results under the condition that the patient remains anonymous (Figure S1). The Informed Consent Form was approved by the Ethics Committee of the “Thalassotherapia-Opatija” Hospital, dated February 10, 2021, No. 01-000-00-17/2-2021. This study adhered to the Helsinki Declaration of the World Medical Association held in Edinburgh, year 2000 [22] and all applicable guidelines aimed to ensure patient safety.

### 2.2. Rheumatological Examination

Rheumatological examination of patients included recording general data (sex and age), medical history (comorbidity and current therapy) from the hospital information system (WinBis, IN2 Ltd., Zagreb, Croatia) and physical examination. Physical examination comprised evaluating SpA activity by determine the sore joint count (SJC) among the 28 and 68 considered for better evaluation of peripheral activity, and the swollen joint count (SwJC) among the 28 considered, duration of morning stiffness measured in minutes, and patient’s self-reported neck/axial pain and SpA activity using a visual analogue scale from 0 to 10 in the past week, as well as the calculation of BASDAI (Bath Ankylosing Spondylitis Disease Activity Index), ASDAS (Axial Spondyloarthritis Disease Activity Score) [22] and the DAPSA (Disease Activity Index in Psoriatic Arthritis) for peripheral disease [23].

### 2.3. Biochemical Laboratory Testing

The biochemical analysis consisted of proinflammatory markers: the erythrocyte sedimentation rate (ESR) [24] and serum C-reactive protein (CRP) responsible for flares [25], anti-streptolysin-O (AST-O), and titer indicating recent infection with group A *Streptococcus bacteria* [26], using a biochemical analyzer [Cobas Pro, Roche Diagnostics, Boehringer Mannheim]. Calprotectin, as a marker of intestinal inflammation and damage [27], was analyzed from stool samples using the immunoturbidimetry method (reagent: Bühlmann fCAL^®^ turbo calprotectin; Bühlmann Laboratories AG, Schönenbuch, Switzerland) on an analyzer (Roche Cobas c501 module; Roche Diagnostics, Mannheim, Germany).

### 2.4. Sampling and Analysis of Saliva

The patient’s unstimulated saliva was collected in the morning hours (until 11 a.m.) by spitting into a sterile conical test tube with a wide opening and cap (50 mL, Falcon, Lawrence, KS, USA), provided that the patient had not consumed food or liquids for at least 3 h prior to sampling according to a previously published protocol [28]. Within 30 min, the saliva samples were centrifuged (350× *g* for 10 min) to separate all cells from the soluble aqueous portion. The aqueous portion was carefully collected by pipet, relocated and stored in sterile conical tubes (2 mL, Falcon, Lawrence, KS, USA) at −20 °C until analysis (no longer than 6 months) of pro-inflammatory substances using the enzyme-linked immunosorbent assay (ELISA) according to the manufacturer’s instructions. Commercially available ELISA kits from Abcam (Cambridge, UK) were used to test undiluted saliva samples for the main outcome variable IL-17A (Cat. No. ab83688) and other variables including IL-12/23 p40 (Cat. No. ab220656), TNF-α (Cat. No. ab46087), and MMP-3 (Cat. No. ab269371). All ELISA measurements were conducted as single determinations, due to the limited availability of sample volume (single saliva collection at the time of TMJ examination) in a single analytical run using the same calibration curve and under strictly controlled experimental conditions to minimize analytical variability. The absorbance was measured at 450 nm (HiPo MPP-96, BioSan, Medical-Biological Research & Technologies, Riga, Latvia) and analyzed using the CurveExpert program (Version 1.40; Copyright © 1995–2009 by Daniel G. Hyams, Hyams Development).

### 2.5. TMJ Examination

A dentist performed an examination of the TMJs according to the DC/TMD protocol [19] and recorded the results in the DC/TMD form, which consists of two axes. Axis I comprises a standardized assessment of patient history and clinical examination and is used to establish physical diagnoses of TMDs. It includes the DC Symptom Questionnaire, which records patient-reported symptoms such as pain location, pain with jaw function, joint sounds, jaw movement limitations, locking episodes, and headache related to jaw activity. The clinical examination involves observation and recording of mandibular movements (range of motion and asymmetry), assessment of joint sounds during mandibular motion, and palpation of lateral poles of the condyles in rest position and during movement to identify familiar joint pain. Axis II consists of a series of questionnaires about the patient’s symptoms, which were filled out by the patient with the doctor’s support.

Axis II represents a series of questionnaires about psychosocial status, pain-related disability, and jaw function, as follows: DC Graded Chronic Pain Scale version (GCPSV) 2.0, Localisation of Pain in Drawings, Jaw Function Limitation Scale (JFLS)-20, Oral Behaviors Checklist (OBC), General Anxiety Disorder (GAD)-7, Patient Health Questionnaire (PHQ)-15: physical symptoms, and Patient Health Questionnaire (PHQ)-9 for depressive disorders [18]. Questionnaires record the characteristics of the disease in detail and provide insight into psychosocial dysfunction (degree of chronic pain, somatization, degree of anxiety, depression, and limitation of mandibular function) [18]. Questionnaires record the characteristics of the disease in detail and provide insight into psychosocial dysfunction (degree of chronic pain, somatization, degree of anxiety, depression, and limitation of mandibular function) [18]. DC/TMD diagnoses were made using Axis I clinical findings and symptom questionnaires according to the Diagnostic Decision Trees [29]. Patients were classified into pain-related TMDs and intra-articular TMJ disorders (IDs) [18,19].

After evaluating the DC/TMD, the patients with SpA were allocated in groups: those with TMJ-ID with intra-articular pain were considered symptomatic (group A); those with TMJ-ID without intra-articular pain were considered asymptomatic (group B), regardless of the presence of clicking, popping, or pain of extra-articular origin; and those without TMDs were considered controls (group C). Individuals with asymptomatic TMJ subluxation were included in group C since it does not involve disc-condyle misalignment, which defines TMJ-ID. Patients without IDs and with a diagnosis from the muscular-related pain and headache group were excluded from the research.

### 2.6. Statistical Analyses

The required sample size was calculated for a Student’s *t*-test of independent samples at the level of statistical significance *p* < 0.05 with a power of statistical analysis of 90% based on the preliminary results (salivary IL-17A cytokine concentration in 24 samples) using “Power analysis” and “Sample size analysis”. The calculation indicated that 25 samples were required for the determination of IL-17A.

Continuous variables with normal distribution were analyzed the using Student’s *t*-test (two groups) and one-way analysis of variance (ANOVA), followed by the Tukey’s post-hoc test (three groups). To reduce the risk of false positive results, the Bonferroni correction was applied. Categorical data were analyzed using the Chi-square tests for multiple independent samples and Yates’s correction, which showed improved accuracy of the *p*-value. Fischer’s exact test precisely determines the *p*-value in the case of small samples or if >20% of the expected results are <5. Correlation matrix analysis was performed between salivary IL-17A concentration and clinical and laboratory parameters of patients. Multiple linear regression was performed to assess whether TMJ-ID predicts IL-17A levels, while controlling for age, gender, and oral parafunctions (OBC questionnaire). Statistical analyses mentioned above were performed using Statistica 14.0.0.15 software (TIBCO, Software Inc., Palo Alto, CA, USA). A receiver operating characteristic (ROC) analysis was performed for salivary IL-17A concentration to assess its ability to discriminate between symptomatic IDs (with arthralgia), and healthy TMJ and between asymptomatic IDs (without arthralgia) and healthy TMJ using MedCalc Statistical Software version 20.011 (MedCalc Software Ltd., Ostend, Belgium). Then, dichotomous groups were formed in respect to the cutoff value. Cutoff value for symptomatic TMJ-ID was calculated among patients of group A, *n* = 64 and group C, *n* = 86 (total *n* = 150), whereas cutoff value for asymptomatic TMJ-ID was calculated among patients of group B, *n* = 50 and group C, *n* = 86 (total *n* = 136). The area under the ROC curve (AUC) with 95% confidence interval (CI) was determined to evaluate diagnostic accuracy. Based on the optimal cutoff values determined, patients were categorised into IL-17A-positive and IL-17A-negative dichotomous groups. Using these cutoff values, sensitivity, specificity, and predictive values (both positive and negative) were calculated. Odds ratios (ORs) were determined based on 2 × 2 contingency tables at the chosen cutoff to further evaluate the diagnostic performance. In all analyses, a *p*-value < 0.05 was considered significant.

## 3. Results

### 3.1. Clinical Characteristics of Patients with SpA

Patients with SpA, who were diagnosed with reactive arthritis (*n* = 62), psoriatic arthritis (*n* = 57), undifferentiated SpA (*n* = 40), HLA-B27+ SpA (*n* = 33), or SpA associated with inflammatory bowel disease (*n* = 8) (data is not displayed graphically) were allocated to three groups as follows: patients with symptomatic TMJ-ID (group A, *n* = 64); patients with asymptomatic TMJ-ID of the TMJ (group B, *n* = 50), and patients without TMDs (group C or control, *n* = 86) (Table 1). Comparison of the clinical characteristics among patients with symptomatic IDs, asymptomatic IDs, and controls is shown in Table 1.

Women comprised 92%, 82%, and 63% of patients in the symptomatic TMJ-ID, asymptomatic TMJ-ID and control groups, respectively, and the number of women was higher in the symptomatic (*p* = 0.0001 and *p* < 0.0001) and asymptomatic TMJ-ID (*p* = 0.0308 and *p* = 0.0140) groups vs. controls using Yates’ correction and the Fischer’s exact test, respectively. Degenerative joint disease (39%) and disc displacement (62.5%) with reduction were more frequently observed in patients with symptomatic TMJ-IDs and asymptomatic TMJ-IDs (32% and 66%, respectively) than in controls (*p* < 0.0001). Disc displacement with reduction and intermittent locking and disc displacement without reduction and with limited opening were more frequently observed in the symptomatic TMJ-ID group (12.5%) than in controls (*p* = 0.0008). Disc displacement without reduction and with limited opening was more frequent in the symptomatic than in the asymptomatic TMJ-ID group (2%, *p* = 0.0384). Disc displacement without reduction and without limited opening and local myalgia did not significantly vary among the groups. Myofascial pain was more frequently observed in symptomatic TMJ-ID (21.8%) and asymptomatic patients (6%) than in controls (*p* < 0.0001 and *p* = 0.0478, respectively). Myofascial pain was also more frequent in symptomatic than asymptomatic TMJ-IDs (*p* = 0.0156). Myofascial pain with referral was more frequent in symptomatic TMJ-IDs (17.2%) and asymptomatic TMJ-IDs (6%) than in controls (*p* < 0.0001 or *p* = 0.0478, respectively). Similarly, headache attributed to TMDs was more frequent in symptomatic TMJ-IDs (14.1%) and asymptomatic TMJ-IDs (10%) than in controls (*p* = 0.0003 or *p* = 0.0059, respectively).

Subluxation was observed at the lowest frequency in patients with symptomatic TMJ-IDs (14%) compared with that in those with asymptomatic TMJ-IDs (50%), (*p* = 0.0001 and *p* < 0.0001) and controls (43%), (*p* = 0.0003 and *p* = 0.0001) using Yates’ correction or Fisher’s exact test, respectively.

Gastrointestinal diseases (gastroesophageal reflux disease, chronic gastritis, fatty liver disease, inflammatory bowel disease, and diverticulosis) were significantly more frequently observed in patients with symptomatic TMJ-IDs (26.6%), (*p* = 0.0184 and *p* = 0.0094) and asymptomatic TMJ-IDs (32%), (*p* = 0.0038 and *p* = 0.0022) vs. control (10.5%) using Yates’ correction or Fisher’s exact test, respectively. A significantly higher number of patients with symptomatic TMJ-IDs (43.8%) than those with asymptomatic TMJ-IDs (22%) had a history of genitourinary infection (*p* = 0.0258, Yates’ correction; *p* = 0.0122, Fisher’s exact test). Treated depression was significantly more frequently observed in patients with symptomatic TMJ-IDs (15.6%) than in controls (2.5%), (*p* = 0.0036). Hyperlipoproteinemia was less frequently observed in patients with symptomatic TMJ-IDs (25%) than in the control group (46.5%), (*p* = 0.0116, Yates’ correction; *p* = 0.0054, Fisher’s exact test), while in those with asymptomatic TMJ-IDs (44%) it was significantly lower using Fisher’s exact test (*p* = 0.0266), but this was only marginally significant (*p* = 0.0530) according to Yates’ correction.

Pain estimated over 30 and 180 days using GCPS-V2.0 score was significantly higher in patients with symptomatic (*p* < 0.0001) and asymptomatic (*p* = 0.0001) TMJ-IDs than in controls using the Bonferroni correction; pain in patients with symptomatic TMJ-IDs was higher than in those with asymptomatic IDs (*p* < 0.0001). Jaw mobility limitations (JFLS-20 Total score) were significantly higher in patients with symptomatic TMJ-IDs than in those with asymptomatic TMJ-IDs and controls (*p* < 0.0001). Oral parafunction (OBC Score), somatization (PHQ-15 Score), and anxiety (GAD-7 Score) were more frequently observed in symptomatic TMJ-IDs than in controls (*p* = 0.0075, *p* < 0.0001, and *p* < 0.0001, respectively). Somatization was more frequent in symptomatic TMJ-IDs than in asymptomatic TMJ-IDs (*p* = 0.0006). No significant difference was observed in the presence of depressive disorders (PHQ-9 Score) among the investigated groups.

The number of sore joints among the 28 joints was higher in patients with symptomatic TMJ-IDs than in controls (*p* = 0.0203). The average age in patients with symptomatic TMJ-IDs was 51 ± 11.4 years (mean ± standard deviation [SD]) and that in the asymptomatic TMJ-ID and control groups was 56 ± 12.2 years, and did not differ significantly (*p* = 0.0563, Bonferroni correction).

### 3.2. Relationship of Salivary IL-17A Concentration and Laboratory Parameters and Patient Symptoms

**Figure 1:**
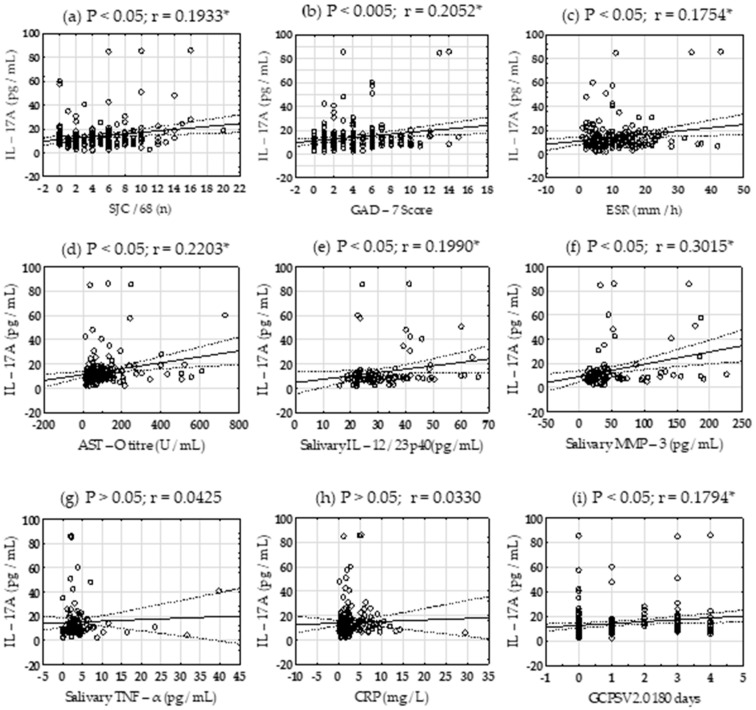
Interrelationship of interleukin (IL)-17A and parameters of SpA activity. Correlation of salivary IL-17A concentration and SJC/68—sore joint count of 68 considered (**a**), GAD-7—General Anxiety Disorder 7 Score (**b**), ESR—erythrocyte sedimentation rate (**c**), serum antistreptolysin-O titer (**d**), salivary IL-12/23 p40 (**e**), salivary MMP-3—matrix metalloproteinase-3 (**f**), salivary TNF-α—tumor necrosis factor-alpha (**g**), CRP—C-reactive protein (**h**), GCPSV 2.0—Graded Chronic Pain Scale version 2.0 over 180 days (**i**). Solid line is fitting line and dashed lines are regression lines. *p* value was lower than 0.05 and it was considered significant (*). Correlation coefficient (r) is shown above scatter plot.

A significant positive correlation (*p* < 0.05) was found between salivary IL-17A concentration and the number of painful joints among the 68 joints considered (r = 0.1933, Figure 1a), GAD-7 Score (r = 0.2052, Figure 1b), ESR (r = 0.1754, Figure 1c), serum AST-O titre (r = 0.2203, Figure 1d), salivary IL-12/23 p40 (r = 0.1990, Figure 1e) and MMP-3 (r = 0.3015, Figure 1f) levels at *p* < 0.05, whereas it did not correlate with the salivary TNF-α concentration (Figure 1g) or CRP levels (Figure 1h). Chronic pain (GCPSV 2.0) over 180 days positively correlated with salivary IL-17A (r = 0.1794, Figure 1i).

Additionally, the increase in salivary IL-17A concentration was followed by the higher SJC/28 (r = 0.1575, Figure S2a), SwJC/28 (r = 0.1413, Figure S2b) and BASDAI (r = 0.1423, Figure S2c), whereas the salivary IL-17A did not correlate with ASDAS (Figure S2d), DAPSA (Figure S2e) and morning stiffness (Figure S2f).

In multiple linear regression, TMJ-ID was a significant predictor of IL-17A when age, gender and oral parafunctions (OBC) were controlled for (R^2^ = 0.151; *p* < 0.001). The unique contribution of TMJ-ID was 12%, while age, gender and parafunctions did not influence IL-17A.

### 3.3. Accuracy of Salivary IL-17A Concentration for the Diagnosis of Symptomatic and Asymptomatic TMJ-ID

**Figure 2:**
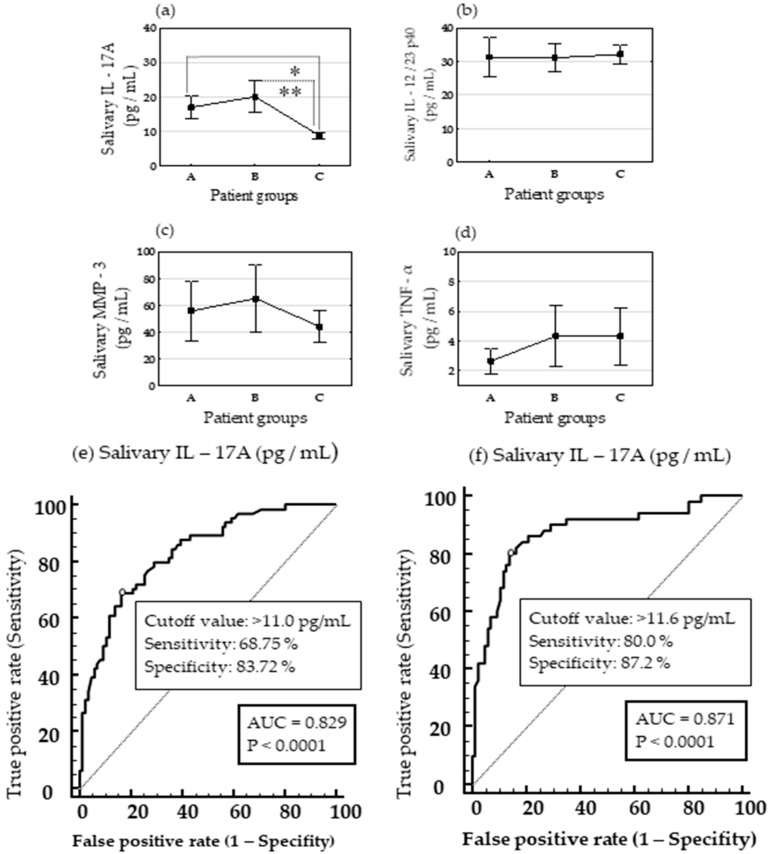
Salivary cytokine concentration and diagnostic accuracy of salivary IL-17A for the diagnosis of temporomandibular joint (TMJ) internal derangements (IDs) in patients with SpA. Graphs show salivary concentration of IL-17A (**a**), IL-12/23 p40 (**b**), MMP-3 (**c**) and TNF-α (**d**) in patients with symptomatic TMJ-IDs (group A), asymptomatic TMJ-IDs (group B) and without TMJ (control, group C). Levels of statistical significance (*p*): * 0.0173; ** < 0.0001 (Bonferroni correction of *p* values obtained by one-way ANOVA and the Tukey post-hoc test). • Mean, and I mean ± 0.95 confidence interval. Plots show the area under the receiver operating characteristics curve (AUC) for salivary IL-17A concentration at the cutoff value of 11 pg/mL for the diagnosis symptomatic TMJ-IDs in patients with SpA (**e**) and 11.6 pg/mL for the diagnosis of asymptomatic IDs in patients with SpA (**f**) with respect to the control. Levels of statistical significance (*p*), AUC, criterions, specificity and sensitivity obtained in receiver operating characteristic analysis are indicated in the plots.

The concentration of IL-17A in saliva (Figure 2a) was significantly higher in patients with symptomatic TMJ-IDs and was 16.9 pg/mL (15.3–18.6) (mean [confidence interval]) and in patients with asymptomatic TMJ-IDs [19.9 pg/mL (17.7–22.3)] compared to the control group [8.8 pg/mL (8.3–9.2)] according to the Bonferroni correction (*p* = 0.0173 and *p* < 0.0001, respectively). The salivary concentrations of IL-12/23 p40 (Figure 2b) and MMP-3 (Figure 2c) were approximately 2–3 times higher than those of IL-17A but did not vary among the groups. Salivary concentration of TNF-α averaged several pg/mL and did not significantly differ among the groups (Figure 2d). In accordance with the differences in the salivary concentration of IL-17A in patients with symptomatic and asymptomatic TMJ-IDs compared to controls, we analyzed the diagnostic accuracy of salivary IL-17A concentration for the diagnosis of symptomatic (Figure 2e) and asymptomatic (Figure 2f) TMJ-IDs using ROC curve analysis. ROC curve analysis (AUC = 0.829, *p* = 0.0001) revealed a salivary IL-17A concentration of 11 pg/mL as the cutoff value for symptomatic TMJ-IDs of TMJs in patients with SpA (group A and group C, *n* = 150), with sensitivity and specificity of 68.75% and 83.72%, respectively (Figure 2e). The positive predictive value of the salivary IL-17A concentration for symptomatic TMJ-IDs was 75.8% (95% CI, 65.4% to 83.9%), and the negative predictive value was 78.2% (95% CI, 71.2% to 83.9%). A salivary IL-17A concentration of 11.6 pg/mL was considered the cutoff value for the diagnosis of asymptomatic TMJ-IDs in patients with SpA (Groups B and C, *n* = 136) in the ROC curve analysis (AUC 0.871, *p* = 0.0001), with a sensitivity of 80% and specificity of 87.2% (Figure 2f). The positive predictive value of the salivary IL-17A concentration for asymptomatic TMJ-IDs was 78.43% (95% CI, 67.3% to 86.5%), and the negative predictive value was 88.2% (95% CI, 81.1% to 92.9%).

### 3.4. Differences in Characteristics of Patients with SpA Based on the Salivary IL-17A 11 pg/mL Cutoff Value

Based on the salivary IL-17A cutoff value of 11 pg/mL for the diagnosis of symptomatic TMJ-IDs, total patients with SpA (*n* = 150, which is the sum of the group A, *n* = 64 and group C, *n* = 86) were classified into two groups: (I) group with salivary IL-17A concentration higher than the cutoff value (*n* = 92) and (II) group with salivary IL-17A concentrations equal to or lower than the cutoff value (*n* = 58), which served as a control in the comparisons (Table 2). Patients with salivary IL-17A concentration > 11 pg/mL exhibited significantly higher frequency of degenerative joint disease (*p* = 0.0002) and disc displacement with reduction (*p* < 0.0001), headache attributed to TMDs (*p* = 0.0023), myofascial pain (*p* = 0.0006), local myalgia (*p* = 0.0209), arthralgia from the group of TMD-related pain and headache (*p* < 0.0001), and less frequent subluxation (*p* = 0.0006), as assessed using Fisher’s exact test. Other TMDs did not differ. Chronic pain over 30 and 180 days (*p* < 0.0001), jaw mobility limitation (JFLS-20 Score, *p* < 0.0001), somatization (PHQ-15 Score, *p* = 0.0054), and anxiety (GASD-7 Score, *p* = 0.0054) were higher in patients with salivary IL-17A concentrations >11 pg/mL than in those with salivary IL-17A concentrations ≤11 pg/mL, as analyzed using the Student’s *t*-test. Oral parafunctions and depression did not differ significantly. The ASDAS was borderline significant (*p* = 0.0573 in Student’s *t*-test); however, DAPSA (*p* = 0.0266), patients’ self-estimated SpA activity (*p* = 0.0239), and axial pain (*p* = 0.0160) were significantly higher in patients with salivary IL-17A concentrations >11 pg/mL. The number of sore and swollen joints and morning stiffness did not significantly differ. Salivary IL-17A concentration (*p* < 0.0001) and AST-O titer (*p* = 0.0016) were higher in the >11 pg/mL group. The mean salivary IL-12/23 p40, MMP-3, and TNF-α concentrations, ESR and CRP did not differ significantly between the groups (Table 2).

### 3.5. Differences in Patients with SpA Based on the Salivary IL-17A Concentration Cutoff Value of 11.6 pg/mL

Patients with SpA were classified into two groups according to the salivary IL-17A cutoff value of 11.6 pg/mL for the diagnosis of asymptomatic TMJ-IDs, as follows: (I) patients with salivary IL-17A concentration higher than the cutoff value (*n* = 84) and (II) patients with salivary IL-17A concentration equal to or lower than the cutoff value (*n* = 52), which served as the control (Table 3). Patients with salivary IL-17A concentration > 11.6 pg/mL exhibited a higher frequency of degenerative joint disease (*p* = 0.0003), disc displacement with reduction (*p* < 0.0001) and headache attributed to TMDs (*p* = 0.0072) according Fisher’s exact test. They also exhibited a increased chronic pain over 30 (*p* = 0.0111) and 180 (*p* = 0.0209) days on the GCPSV scale, PHQ-15 scores for physical symptoms (*p* = 0.0255), ASDAS (*p* = 0.0052), patient’s self-estimated SpA activity (*p* = 0.0112), axial pain intensity (*p* = 0.0001) and IL-17A (*p* < 0.0001), according to the Student’s *t*-test, vs. control. The other TMDs, JFLS, OBC, GAD-7 and PHQ-9 scores, DAPSA, number of sore and swollen joints, morning stiffness, salivary IL-12/23 p40, MMP-3, TNF-α, serum AST-O titer, ESR, and CRP did not significantly vary between the compared groups (Table 3).

## 4. Discussion

This study demonstrates for the first time that salivary IL-17A concentrations are higher in patients with SpA with TMJ-IDs—both currently symptomatic and asymptomatic—than in controls. The average age of participants in this study exceeded 50 years, and it was not always possible to conclude how long TMD lasted, especially in asymptomatic patients. The duration of SpA in the same patients ranges from 18 to 26 years [30]. However, the onset of active disease was statistically significantly earlier (about the age of 30 years) in patients with symptomatic TMJ-ID compared to controls [30], which corresponds to the reproductive age in women, who dominate among the patients in this study and in the general population with TMJ-IDs [31]. In addition, there is increasing evidence that TMJ-ID is a sign of clinically and biologically active SpA, which appears in young adults [30].

Dysbiosis strongly affects DC functions, which are specialized to recognize, internalize, process, and present foreign antigens to T cells and to skew the immune response, although they represent a tiny leukocyte subpopulation [9]. DC-originated free p40 combines with p19 to form IL-23, which is indispensable for the differentiation of the T helper (Th) 17 immune response in the oral/gut mucosa [6]. IL-23, together with IL-1α, also originating from DCs, strongly activates ILC3, which leads to further secretion of IL-17A in the intestinal mucosa [3,7,8]. Moreover, activated DCs [9] and ILC3 [10] leave the digestive tract and spread via lymph and blood vessels toward the uvea, entheses and joints of patients with SpA [7]. Patients with r-axSpA and PsA have pronounced vascular proliferation and perivascular infiltration of inflammatory cells and fibrosis in soft tissues [11]. In extraintestinal tissues, ILC3/Th17 crosstalk promotes the synthesis of IL-17A, which further stimulates TNF-α and IL-22 with strong inflammatory properties in joints and entheses, and causes local pain [7,8].

Salivary IL-17A levels directly correlate with chronic TMJ pain over 180 days, regardless of the current pain during clinical TMJ examination, taking into account the nature of the pain which may appear and disappear. It indicates that salivary IL-17A might have the characteristic of being a valuable pro-inflammatory marker for TMJ-IDs, considering its central role in SpA pathophysiology [2,7,32,33], in accordance with recent findings that both TMJ pain and joint sounds are reliable predictors of TMD severity [34] and axial SpA [30]. Patients with SpA involving the TMJs exhibit greater scintigraphic uptake in the TMJs than healthy controls, confirming a marked inflammatory process in SpA [35]. In this study, higher salivary IL-17A concentration was associated with axial SpA exacerbation, reflecting in higher patients’ estimated activity of SpA, axial pain or ASDAS values. Salivary IL-17A concentrations also positively correlated with the SJC and SwJC among the 28 examined, as well as SJC of the 68 patients considered, and indicates activation of the peripheral form of SpA [32]. Higher DAPSA was found in patients with salivary IL-17A concentration higher than 11 pg/mL. Pain in SpA has shown a significant association with infections [32] and the family *Streptococcaceae* in intestinal microbiota [5]. Accordingly, patients with symptomatic and asymptomatic TMJ-IDs showed increased frequency of gastro-intestinal disease than control, and salivary IL-17A concentrations significantly positively correlated with serum AST-O titer and ESR. Moreover, patients with symptomatic TMJ-IDs more frequently recorded genitourinary infections in their medical history than patients with asymptomatic IDs, suggesting their role in SpA exacerbation.

In patients with SpA, salivary IL-17A concentrations positively correlated with IL-12/23 p40, an early proinflammatory indicator of DC activity [6], and MMP-3, a marker of extracellular matrix remodelling in radiographic axial SpA during exacerbation [13]. These findings support the concept of an oral–intestinal–joint axis in the pathogenesis of SpA [10], and the efficacy of IL-23 and IL-12/23 p40 inhibitors as first-line biologic treatments for patients in the early phase of PsA [36], which was frequently represented in our study.

BASDAI, but not ASDAS, correlated positively with salivary IL-17A, although both are approved and recommended for assessing axial SpA activity [37]. TNF-α correlates with BASDAI [33]; it supports CRP production in r-axSpA [38]. CRP is an important determinant of ASDAS, which makes ASDAS a slightly more objective instrument then BASDAI [37]. CRP also makes the DAPSA a reliable instrument for assessing peripheral PsA activity [39], which does not depend on salivary IL-17A levels. Moreover, salivary IL-17A correlated neither with CRP, nor with salivary TNF-α, in patients with SpA and represented an independent predictor for TMD-IDs, irrespective of age, gender and OBC, according the fact that IL-17 efficiently distinguished patients with SpA and controls [33]. Cytokine concentrations may vary across anatomical sites depending on the stage of SpA [40]. TNF-α is increased in peripheral blood of patients with SpA in active disease vs. controls [38] and is abundantly expressed in the synovial fluid of the TMJs and promotes inflammation at TMJ sites [15].

Various ID subtypes with intracapsular pain were substantially represented among patients with SpA and symptomatic TMJ-IDs, whereas only disc displacement with reduction and degenerative joint disease were more frequent in patients with SpA and asymptomatic TMJ-IDs than in controls, confirming a more extensive TMJ disorder in symptomatic patients [41]. Myofascial pain, with or without referral, and headache attributed to TMDs coincided with both symptomatic and asymptomatic TMJ-IDs and were likely associated with chronic pain in patients with TMDs [42]. Beyond pain, active axSpA imposes a substantial psychological burden in the form of somatization, characterised by physical symptoms without clear organic causes [43]. Somatization was higher in the symptomatic TMJ-ID group and likely contributed to anxiety and depression [44], as well as decreased lower jaw mobility, more frequent subluxation, and parafunctional habits [31,45,46]. In this study, anxiety significantly positively correlated with salivary IL-17A concentration, consistent with evidence that IL-17A levels increase in circulation and the brain during anxiety [47] and depression [48]. Furthermore, significantly more patients with symptomatic TMJ-IDs were treated for depression than controls, confirming the greater prevalence of depression with increased SpA activity [46].

In clinical practice, patients with asymptomatic TMJ-IDs who do not experience pain are typically not referred for radiological examination, for example MRI, which represents the “gold standard” for structural disc abnormalities regardless of symptoms [16]. The DC/TMD protocol is validated for diagnosing symptomatic TMJ-IDs, but not asymptomatic ones [19]; hence, the latter may often remain undetected. Therefore, additional practical diagnostic tools based on the inflammatory nature of TMDs are warranted [49]. In this study, we demonstrated that salivary IL-17A, owing to its inflammatory properties, exhibits diagnostic accuracy for both currently symptomatic (cutoff: 11 pg/mL) and currently asymptomatic (cutoff: 11.6 pg/mL) TMJ-IDs, with high sensitivity and specificity. No one has proven so far that salivary IL-17A possesses diagnostic accuracy for TMJ-IDs in patients with SpA and that salivary IL-17A correlates with particular clinical and laboratory SpA activity, although saliva composition may vary in immunocompromised patients due to dryness of the oral mucosa [30]. Notably, patients with SpA and salivary IL-17A concentrations >11 pg/mL showed a significantly higher prevalence of DD with reduction and degenerative joint disease accompanied by intracapsular pain, headache attributed to TMD, myofascial pain, local myalgia, anxiety, and somatization—all consistent with symptomatic TMJ-IDs [45]—compared with those with IL-17A concentrations ≤11 pg/mL. Likewise, patients with SpA and IL-17A concentration >11.6 pg/mL (the asymptomatic ID cutoff) demonstrated a higher frequency of disc displacement with reduction, degenerative joint disease, headache attributed to TMD, and somatization, indicating complex psychophysical interactions even in patients with currently asymptomatic IDs [43,49]. Patients with SpA and asymptomatic TMJ-ID had higher frequencies of acute axial SpA and chronic inflammatory sacroiliac joint remodelling compared to the controls [30], which is mediated by IL-17A [7]. Therefore, IL-17A can be increased in asymptomatic and symptomatic patients with TMJ-IDs, who have both increased axial disease activity and clinical burden. Besides headache attributed to TMD, somatization may explain the greater chronic pain severity reported in patients with asymptomatic TMJ-IDs than in controls, as recently confirmed in r-axSpA [50].

This study has some limitations, which partly stem from a complex research topic. Patients with SpA who were receiving biologic or targeted synthetic DMARDs were excluded, because these drugs inhibit the effects of cytokines, and we could not examine the influence of cytokines on disease activity, but it is possible that they cause selection bias, alongside the cross-sectional design of this study. In this investigation, patients were sequentially recruited from the rheumatology outpatient clinic. Consequently, women predominated across all three groups, although psoriatic arthritis affects both sexes equally, and r-axSpA is more prevalent in men [51]. Women are generally more sensitive to pain, which is a characteristic of SpA, and are more likely to seek medical help [51], although they exhibit a higher incidence of TMDs in reproductive age [31]. In future research, a prospective cohort design is preferred with careful adjustment of the study population by gender, and saliva collection should be standardized as much as possible. Hence, careful selection of participants in other studies is warranted for appropriate comparison. Moreover, all SpA subtypes were included in this study, as they share many clinical features yet differ in certain aspects [3]. Therefore, comparisons with studies focusing on single SpA subtypes or different subtype ratios should be interpreted cautiously. As this investigation was conducted at a single center, multicenter, MRI-based studies with larger sample sizes, particularly focusing on single SpA, are recommended for better representation and reduction of possible selection bias.

## 5. Conclusions

IL-17A, a key cytokine in SpA immunopathogenesis [7], is readily detectable in saliva and may serve as a laboratory marker of SpA activity associated with both currently symptomatic and asymptomatic TMJ-ID conditions. Salivary IL-17A demonstrated comparable diagnostic accuracy for currently symptomatic and asymptomatic TMJ-IDs based on similar cutoff values, and it was connected with TMD severity in patients with SpA. These findings suggest that both asymptomatic and symptomatic TMJ-IDs in patients with SpA warrant equal clinical attention and the application of MRI when indicated [16], emphasizing anti-inflammatory and patient-centerd TMD management. Furthermore, emerging evidence [49] highlights the potential therapeutic benefits of biological agents targeting IL-17A in patients with SpA and TMJ-IDs.

## Figures and Tables

**Table 1:** Clinical findings in patients with spondyloarthritis (SpA). Differences among groups of patients with symptomatic temporomandibular joint (TMJ) internal derangement (ID) (group A), asymptomatic TMJ-ID (group B) and patients with no TMD (group C, controls) were analyzed.

TMDs	Group A *n* = 64	Group B *n* = 50	Group C *n* = 86	Fisher’s Exact Test	χ^2^ Test for Multiple Samples	Yates’ Correction
	*n* (%)			*p* Levels		
Sex/female	59 (92)	41 (82)	54 (63)	<0.0001 ^a^ 0.0140 ^b^	<0.0001	<0.0001 ^a^; 0.0001 ^y^ 0.0186 ^b^; 0.0308 ^y^
Degenerative joint disease	25 (39)	16 (32)	0 (0)	<0.0001 ^a^ <0.0001 ^b^		
DD with reduction	40 (62.5)	33 (66)	0 (0)	<0.0001 ^a^ <0.0001 ^b^		
DD with reduction and intermittent locking	8 (12.5)	2 (4)	0 (0)	0.0008 ^a^		
DD without reduction and with limited opening	8 (12.5)	1 (2)	0 (0)	0.0008 ^a^ 0.0384 ^c^		
DD without reduction and without limited opening	3 (4.6)	0 (0)	0 (0)	0.0756 ^a^		
Local myalgia	3 (4.7)	0 (0)	0 (0)	0.0756 ^a^		
Myofascial pain	14 (21.8)	3 (6)	0 (0)	<0.0001 ^a^ 0.0478 ^b^ 0.0156 ^c^		
Myofascial pain with referral	11 (17.2)	3 (6)	0 (0)	<0.0001 ^a^ 0.0478 ^b^		
Headache attributed to TMDs	9 (14.1)	5 (10)	0 (0)	0.0003 ^a^ 0.0059 ^b^		
Arthralgia	64 (100)	0 (0)	0 (0)	<0.0001 ^a^ <0.0001 ^c^		
Subluxation	9 (14)	25 (50)	37 (43)	0.0001 ^a^ <0.0001 ^c^	0.0001	0.0001 ^a^; 0.0003 ^y^ <0.0001 ^c^; 0.0001 ^y^
Gastro-intestinal disease	17 (26.6)	16 (32)	9 (10.5)	0.0094 ^a^ 0.0022 ^b^	0.0050	0.0100 ^a^; 0.0184 ^y^ 0.0018 ^b^; 0.0038 ^y^
Genito-urinary infections in anamnesis	28 (43.8)	11 (22)	24 (27.9)	0.0122 ^c^	0.0294	0.0151 ^c^; 0.0258 ^y^
Treated depression	10 (15.6)	3 (6)	2 (2.5)	0.0036 ^a^	0.0083	0.0030 ^a^; 0.0077 ^y^
Hyperlipoproteinemia	16 (25.0)	22 (44)	40 (46.5)	0.0054 ^a^ 0.0266 ^c^	0.0199	0.0071 ^a^; 0.0116 ^y^ 0.0327 ^c^; 0.0530 ^y^
				ANOVA	Tukey’s test	Bonferonni correction
	Mean ± SD			*p* Levels		
GCPSV 2.0 30 days (Score)	2.8 ± 1.0	0.8 ± 1.4	0.1 ± 0.3	<0.0001	<0.0001 ^a^ <0.0001 ^b^ <0.0001 ^c^	<0.0001 ^a^ 0.0001 ^b^ <0.0001 ^c^
GCPSV 2.0 180 days (Score)	2.7 ± 1.0	0.7 ± 1.4	0.1 ± 0.3	<0.0001	<0.0001 ^a^ <0.0001 ^b^ <0.0001 ^c^	<0.0001 ^a^ 0.0002 ^b^ <0.0001 ^c^
JFLS-20 Total score	0.8 ± 0.7	0.1 ± 0.2	0.0 ± 0.2	<0.0001	<0.0001 ^a^ <0.0001 ^c^	<0.0001 ^a^ <0.0001 ^c^
OBC Score	19.8 ± 9.3	17.4 ± 7.8	15.9 ± 6.0	0.0103	0.0062 ^a^	0.0075 ^a^
PHQ-15 Score	8.0 ± 4.02	5.7 ± 3.1	4.9 ± 2.7	<0.0001	<0.0001 ^a^ 0.0004 ^c^	<0.0001 ^a^ 0.0006 ^c^
GAD-7 Score	5.8 ± 3.6	4.3 ± 3.2	2.9 ± 3.2	<0.0001	<0.0001 ^a^	0.0001 ^a^
PHQ-9 Score	4.1 ± 2.5	3.9 ± 2.6	3.1 ± 3.3	0.0702		
Sore joint count/28	3.1 ± 4.0	2.9 ± 3.0	1.7 ± 2.3	0.0108	0.0170 ^a^	0.0203 ^a^
Age (year)	51.0 ± 11.4	56.0 ± 12.2	56.0 ± 12.2	0.0318	0.0468 ^a^	0.0563 ^a^

Statistical significance (*p*) lower than 0.05 was considered significant. *p* between group A and group C (^a^); group B and group C (^b^); group A and group B (^c^); Yates’ correction (^y^). Abbreviations: DD—disc displacement; GAD-7—General Anxiety Disorder 7; GCPSV 2.0—Graded Chronic Pain Scale version 2.0; JFLS-20—Jaw Function Limitation Scale 20; OBC—Oral Behaviors Checklist; PHQ-9—Patient Health Questionnaire 9; PHQ-15—Patient Health Questionnaire 15; TMJ—temporomandibular joint.

**Table 2:** Comparison of TMDs: clinical and laboratory activity of SpA in patients classified in groups with respect to the cutoff IL-17A concentration (11 pg/mL) for diagnosis of symptomatic TMJ-ID.

Patients with SpA	Interleukin-17A		Fisher’s Exact Test
	>11 pg/mL, *n* = 92	≤11 pg/mL, *n* = 58	
	*n* (%)	*n* (%)	*p* Levels
Degenerative joint disease	19.0 (32.75)	8.0 (8.69)	0.0002 *
DD with reduction	27.0 (46.55)	11.0 (11.95)	0.0000 *
DD with reduction and intermittent locking	4.0 (6.89)	4.0 (4.34)	0.3728
DD without reduction and with limited opening	5.0 (9.43)	3.0 (3.26)	0.1473
DD without reduction and without limited opening	2.0 (3.44)	1.0 (1.08)	0.3318
Headache attributed to TMDs	8.0 (13.79)	1.0 (1.08)	0.0023 *
Myofascial pain	11.0 (18.96)	2.0 (2.17)	0.0006 *
Myofascial pain with referral	7.0 (13.72)	4.0 (4.34)	0.0759
Local myalgia	04.0 (7.40)	0.0 (0.00)	0.0209 *
Arthralgia	42.0 (72.41)	20.0 (21.73)	0.0000 *
Subluxation	13.0 (22.41)	46.0 (50.00)	0.0006 *
	Mean ± SD	Mean ± SD	Student’s *t* test (*p* levels)
GCPSV 2.0 30 days	1.2 ± 1.5	0.7 ± 1.33	<0.0001 *
GCPSV 2.0 180 days	1.2 ± 1.5	0.7 ± 1.3	<0.0001 *
JFLS-20 Score	0.4 ± 0.6	0.2 ± 0.4	<0.0001 *
OBC Score	17.5 ± 7.8	117.4 ± 6.7	0.8238
PHQ-15 Score	6.2 ± 3.7	5.6 ± 3.3	0.0054 *
GAD-7 Score	4.5 ± 3.8	3.8 ± 3.7	0.0054 *
PHQ-9 Score	3.5 ± 3.1	3.2 ± 3.0	0.1414
ASDAS	2.2 ± 0.8	1.9 ± 0.7	0.0573
DAPSA	19.4 ± 11.0	12.6 ± 8.2	0.0266 *
Activity of SpA (VAS 1–10)	4.4 ± 2.3	3.5 ± 2.0	0.0239 *
Axial pain (VAS 1–10)	4.6 ± 2.7	3.6 ± 2.2	0.0160 *
Sore joint number/28	2.8 ± 4.0	2.0 ± 2.6	0.1375
Swollen joint number/28	0.6 ± 1.9	0.4 ± 0.8	0.2697
Morning stiffness (minutes)	1.7 ± 1.7	1.6 ± 1.6	0.5925
Salivary IL-17A (pg/mL)	19.4 ± 13.0	7.7 ± 2.1	<0.0001 *
Salivary IL-12/23 p40 (pg/mL)	36.4 ± 13.7	30.8 ± 10.8	0.0895
Salivary MMP-3 (pg/mL)	57.6 ± 55.3	45.4 ± 44.6	0.3580
Salivary TNF-α (pg/mL)	3.2 ± 2.8	3.9 ± 5.7	0.6585
Serum antistreptolysin-O (U/mL)	136.7 ± 152.3	77.5 ± 56.7	0.0016 *
ESR (mm/h)	10.4 ± 6.7	10.6 ± 7.7	0.9026
C-reactive protein (mg/L)	2.4 ± 1.9	2.8 ± 3.9	0.4802
Fecal calprotectin (μg/g)	95.9 ± 130.8	99.9 ± 152.9	0.8896

Number of patients (*n*) in the group with IL-17A above 11 pg/mL, *n* = 92 and in the group with IL-17A equal to or lower than 11 pg/mL, *n* = 58. Statistical significance (*p*) lower than 0.05 was considered significant (*). Abbrevations: ASDAS—Ankylosing Spondylitis Disease Activity Score; DAPSA—Disease Activity Index for Psoriatic Arthritis; DD—disc displacement; ESR—erythrocyte sedimentation rate; GAD-7—General Anxiety Disorder 7; GCPSV 2.0—Graded Chronic Pain Scale version 2.0; ID—internal derangement; JFLS-20—Jaw Function Limitation Scale 20; MMP-3—matrix metalloproteinase-3; OBC—Oral Behaviors Checklist; PHQ-9—Patient Health Questionnaire 9; PHQ-15—Patient Health Questionnaire 15; SpA—spondyloarthritis, TMD—temporomandibular disorders; TNF-α—tumor necrosis factor-alpha; VAS—visual analogue scale.

**Table 3:** Comparison of TMDs: clinical and laboratory activity of SpA in patients classified in groups with respect to the cutoff IL-17A concentration (11.6 pg/mL) for diagnosis of asymptomatic TMJ-ID.

Patients with SpA	Interleukin-17A		Fisher’s Exact Test
	>11.6 pg/mL, *n* = 84	≤11.6 pg/mL, *n* = 52	
	*n* (%)	*n* (%)	*p* Levels
Degenerative joint disease	13.0 (25)	3.0 (3.57)	0.0003 *
DD with reduction	27.0 (51.92)	6.0 (7.16)	<0.0001 *
DD with reduction and intermittent locking	2.0 (4.0)	0.0 (0.0)	0.1444
DD without reduction and with limited opening	0.0 (0.0)	1.0 (1.19)	0.6176
DD without reduction and without limited opening	0.0 (0.0)	0.0 (0.0)	-
Headache attributed to TMDs	5.0 (9.61)	0.0 (0.0)	0.0072 *
Myofascial pain	2.0 (4.0)	1.0 (1.19)	0.3255
Myofascial pain with referral	2.0 (4.0)	1.0 (1.19)	0.3255
Local myalgia	0.0 (0.0)	0.0 (0.0)	-
Arthralgia	0.0 (0.0)	0.0 (0.0)	-
Subluxation	28.0 (53.8)	44.0 (52.4)	0.5045
	Mean ± SD	Mean ± SD	Student’s *t* test (*p* levels)
GCPSV 2.0 30 days	0.6 ± 1.3	0.2 ± 0.6	0.0111 *
GCPSV 2.0 180 days	0.6 ± 1.2	0.2 ± 0.7	0.0209 *
JFLS-20 Score	0.1 ± 0.2	0.03 ± 0.15	0.2651
OBC Score	16.4 ± 8.2	16.5 ± 5.6	0.9355
PHQ-15 Score	5.9 ± 2.8	4.8 ± 2.8	0.0255 *
GAD-7 Score	4.2 ± 3.3	3.6 ± 3.7	0.2864
PHQ-9 Score	3.8 ± 2.8	3.1 ± 3.3	0.2043
ASDAS	2.2 ± 0.7	1.9 ± 0.7	0.0052 *
DAPSA	14.5 ± 6.1	12.5 ± 8.4	0.3237
Activity of SpA (VAS 1–10)	4.2 ± 2.1	3.3 ± 2.0	0.0112 *
Axial pain (VAS 1–10)	5.0 ± 2.5	3.5 ± 2.0	0.0001 *
Sore joint count/28 (n)	2.7 ± 2.9	1.9 ± 2.4	0.0772
Swollen joint count/28 (n)	0.7 ± 1.8	0.4 ± 0.8	0.1937
Morning stiffness (minutes)	16.9 ± 24.4	20.5 ± 32.6	0.5254
Salivary IL-17A (pg/mL)	21.3 ± 15.6	7.7 ± 2.2	<0.0001 *
Salivary IL-12/23 p40 (pg/mL)	31.3 ± 9.2	31.5 ± 10.1	0.9038
Salivary MMP-3 (pg/mL)	61.7 ± 53.4	46.5 ± 46.5	0.2387
Salivary TNF-α (pg/mL)	4.4 ± 6.6	4.2 ± 6.0	0.6834
Serum antistreptolysin O (U/mL)	120.7 ± 129.2	84.8 ± 88.8	0.0762
ESR (mm/h)	13.3 ± 8.9	10.9 ± 7.9	0.9179
C-reactive protein (mg/L)	3.0 ± 2.8	3.0 ± 4.3	0.4915
Fecal calprotectin (μg/g)	98.6 ± 124.2	95.1 ± 132.1	0.8509

Statistical significance (*p*) lower than 0.05 was considered significant (*). Abbreviations: ASDAS—Ankylosing Spondylitis Disease Activity Score; DAPSA—Disease Activity Index for Psoriatic Arthritis; DD—disc displacement; ESR—erythrocyte sedimentation rate; GAD-7—General Anxiety Disorder 7; GCPSV 2.0—Graded Chronic Pain Scale version 2.0; ID—internal derangement; JFLS-20—Jaw Function Limitation Scale 20; MMP-3—matrix metalloproteinase-3; OBC—Oral Behaviors Checklist; PHQ-9—Patient Health Questionnaire 9; PHQ-15—Patient Health Questionnaire 15; SpA—spondyloarthritis, TMD—temporomandibular disorders; TNF-α—tumor necrosis factor-alpha; VAS—visual analogue scale.

## Data Availability

Data from the present study are available from the corresponding author upon reasonable formal request.

## Supplementary Materials

The following supporting information can be downloaded at: https://www.mdpi.com/article/10.3390/biomedicines14020424/s1.

## Abbreviations

ANOVA	One-way analysis of variance
ASDAS	Ankylosing Spondylitis Disease Activity Score
AUC	Area under the receiver operating characteristics curve
BASDAI	Bath Ankylosing Spondylitis Disease Activity Index
CI	Confidence interval
CRP	C-reactive protein
DAPSA	Disease Activity Index for Psoriatic Arthritis
DCs	Dendritic cells
DC/TMD	Diagnostic Criteria for Temporomandibular Disorders
DD	Disc displacement
ESR	Erythrocyte sedimentation rate
GAD-7	General Anxiety Disorder 7
GCPSV 2.0	Graded Chronic Pain Scale version 2.0
ID	Internal derangement
IL	Interleukin
ILC3	Innate lymphoid cells 3
IL12/23 p40	Free common protein 40 subunit of interleukin -12 and IL-23
JFLS-20	Jaw Function Limitation Scale 20
MMP-3	Matrix metalloproteinase-3
m.o.	Microorganism
MRI	Magnetic resonance imaging
n	Number of patients
OBC	Oral Behaviors Checklist
OR	Odds ratio
PHQ-9	Patient Health Questionnaire 9
PHQ-15	Patient Health Questionnaire 15
ROC	Receiver operating characteristic curve
SJC	Sore joint count
SwJC	Swollen joint count
SpA	Spondyloarthritis
Th	T helper
TMD	Temporomandibular disorder
TMJ	Temporomandibular joint
TNF-α	Tumor necrosis factor-alpha
VAS	Visual analogue scale
