# Supplementary material for: Diagnostic Accuracy of Interleukin-17A for Internal Derangements of Temporomandibular Joints in Patients with Spondyloarthritis
Source: Biomedicines. 2026 Feb 13;14(2):424. doi: 10.3390/biomedicines14020424 (PMC12938568; doi:10.3390/biomedicines14020424)
Supplement: Supplementary file 1 [file biomedicines-14-00424-s001.zip › Figure legends for Supplementary files.pdf]

Figure legends for supplementary files

Figure 1S.

Clinical protocol for the recruitment of patients with spondyloarthritis (SpA) in the research. Abbreviations: DC/TMD – Diagnostic Criteria for Temporomandibular Disorders; n – number of patients; TMJ – temporomandibular joint, SpA – spondyloarthrytis.

Figure 2S.

Interrelationship of interleukin (IL)-17A and additional clinical parameters of SpA activity.
Correlation of salivary IL-17A concentration and SJC/28 - sore joint count of 28 consid-ered (a); SwJC/28 - swollen joint count of 28 considered (b); BASDAI - Bath Ankylosing Spondylitis Disease Activity Indeks (c); ASDAS - Axial Spondy-loarthritis Disease Ac-tivity Score (d); DAPSA - Disease Activity Index in Pso-riatic Arthritis (e) and morning stiffness (f). Significance (P) lower than 0.05 was considered significant (*). P value was lower than 0.05 and it was considered significant (*). Correlation coefficient (r) is shown above dot plot.
